# Fabrication of Au Nanorods by the Oblique Angle Deposition Process for Trace Detection of Methamphetamine with Surface-Enhanced Raman Scattering

**DOI:** 10.3390/s19173742

**Published:** 2019-08-29

**Authors:** Baini Li, Tianran Wang, Qingqing Su, Xuezhong Wu, Peitao Dong

**Affiliations:** College of Intelligence Science and Technology, National University of Defense Technology, Changsha 410073, China

**Keywords:** oblique angle deposition (OAD), surface-enhanced Raman scattering (SERS), methamphetamine, Au nanorods

## Abstract

Oblique angle deposition (OAD) is a simple, low cost, effective, and maskless nanofabrication process. It can offer a reliable method for the mass fabrication of uniform metal nanorods which can be used as the surface-enhanced Raman scattering (SERS) substrate with an excellent enhancing performance. Up to now, Ag nanorods SERS substrates have been extensively studied. However, Ag is chemically active and easy to oxidize under atmospheric conditions. Comparatively, Au is chemically stable and has better biocompatibility than Ag. In this paper, we in detail, studied the electromechanical (EM) field distribution simulation, fabrication, and application of Au nanorods (AuNRs) on trace detection of methamphetamine. According to the finite-difference time-domain (FDTD) calculation results, the maximum EM intensity can be obtained with the length of AuNRs to be 800 nm and the tilting angle of AuNRs to be 71° respectively. The aligned Au nanorod array substrate was fabricated by the OAD process. The two key process parameters, deposition angle, and deposition rate were optimized by experiments, which were 86° and 2 Å/s, respectively. Using 1,2-bis (4-pyridyl) ethylene (BPE) as the probe molecule, the limit of detection (LOD) was characterized to be 10^−11^ M. The AuNRs were also used to detect methamphetamine. The LOD can be down to M (i.e., 14.92 pg/ml), which meet the requirements of the on-site rapid detection of the methamphetamine in human urine (500 ng/ml).

## 1. Introduction

Surface-enhanced Raman scattering (SERS) has been widely used as a powerful tool for ultrasensitive chemical analysis [1] and bioanalysis [2]. It can be used in the area of food safety [3], environmental monitoring [4], early disease diagnosis [5], drug detection [6], etc. A key role in SERS performances is played by the substrate. Panneerselvam et al. [7] proposed to divide the SERS substrates insto two types: solid substrate, which is mainly made of metallic nanostructures on a solid-state silicon or glass chip and colloidal substrate, which is mainly formed of metal nanoparticles. The preparation process of the colloid substrate is simple and the SERS performance is excellent. However, the size and morphology of the nanoparticles are difficult to control. Furthermore, the nanoparticles are easy to aggregate in the solution. In addition, the metal nanoparticle solution may fail due to long-term storage or vibration. These will result in a non-uniform and non-stable SERS performance and make it difficult for quantitative analysis using the SERS technique. In comparison, the solid-state SERS substrate has better uniformity and repeatability of the size and morphology of the nanostructures, which is inherited from the advantages of the fabrication process. These will lead to a better SERS performance; the solid-state SERS substrate also has a long shelf life (1 year at least) [7]. 

The electrochemically roughened Ag electrode was the first substrate applied to SERS, which had poor SERS efficiency [8]. After that, researchers focused on how to make high-performance SERS solid-state substrates. The comparison of several typical methods is listed in Table 1. Unfortunately, many of previous methods proposed were complex, high cost, and time consuming, which made it difficult for mass production [9]. Porous anodic alumina oxide (AAO) can only control the nanostructure parameters by adjusting the template structure. Thus, the adjustment capability is restricted. The process of nanosphere lithography (NSL) is complicated and difficult to control. Electron beam lithography (EBL) can fabricate more complicated nanostructures, such as nanotubes, which provide the hollow structure to store or transport molecules. However, EBL costs a lot and is time consuming, which is not suitable for mass production. As for the self-assembly method, it is difficult to precisely control the morphology of the ultimate structures. Oblique angle deposition (OAD) is a physical vapor deposition method in which the normal of the substrate surface is at a large oblique angle (>75°) to the moving direction of evaporated atoms. Through making use of the shadowing effect, the OAD can easily fabricate the nanostructure in a maskless manner. The nanorods can be fabricated in only one step. In conclusion, OAD is a very promising SERS substrate fabrication method that features the advantages of easy implementation, more control, low cost, less time consuming, and suitable for mass production.

Nobel metals, such as Ag and Au, are highly preferred for SERS substrate fabrication because of their electronic structure, surface morphology, and interactions with target analytes [7]. So far, Au and Ag nanostructure arrays fabricated by OAD have been applied in SERS, extensively. Zhao et al. pioneered the work of preparing AgNRs structure [41,42,43]. The enhancement factor of AgNRs substrate can reach 10^8^. Numerous other groups had optimized the performance of AgNRs substrate by adjusting the process parameters [44,45,46]. In recent years, more methods based on OAD have been investigated to fabricate novel SERS substrate for improving SERS performance, reducing costs, etc [47]. Singh et al. demonstrated that if AgNRs grew at 140 K, it would get the same length of nanorods as those done at room temperature in a lower deposition time and show greater SERS performance than the substrate made at room temperature [48]. However, Oh et al. got the opposite conclusion that substrates made at higher temperatures had higher SERS enhancements [49]. There is little in the literature that studies this temperature dependence with other nanostructures, such as AuNRs; perhaps this will be a new area that will be expanded on in the near future for higher SERS performance [47]. For fabrication into smaller dimensions with better control, template assisted deposition was investigated. Templates are surfaces with defined surface structures, so when used in OAD, there will be predefined shadowing [47]. Lithography [50], self-assembled nanoparticles [51], and nano-imprinted polymers [52] are typically used for making templates. However, even with the improved order of nanostructures by templates, the SERS reproducibility remains an issue that needs further study [53]. So far, the tilted nanorod substrate is still of great potential for the application to SERS, which is not only high SERS performance, but also one-step deposition process.

The electromagnetic (EM) field distribution simulation, optimization of structure (e.g., rod length), deposition angle, underlying film thickness, and other work of AgNR substrate have been fully completed. However, a tricky problem exists that once the AgNRs are exposed to ambient environments. It is susceptible to surface oxidation and contamination. By contrast, Au is more chemically stable than Ag, which can be stored for a long period of time without oxidation [54]. Moreover, Au has a better biocompatibility, which can physically adsorb or chemically bond with DNA or protein. Zhao et al. [55] fabricated SiNRs array and coated with an Au layer by sputtering. The LOD for 1,2-bis (4-pyridyl) ethylene (BPE) was 10^−7^ M. Using BPE with the concentration of 10^−4^ as a probe molecule, they also found that the Raman signal on Si nanorods with Au coating samples stored under room conditions for 1–3 years remains strong. This proved that Au is a kind of material that has prolonged shelf time for SERS applications. We can see that the sensitivity of SiO_2_@Ag NRs needs further improvement, which maybe indicates that the Raman signal enhancing capability of SiO_2_ cores is not as good as Au or Ag. Yi-Jun Jen at al. utilized one-step glancing angle deposition to fabricate gold and silver nanohelix arrays (NHAs) on smooth glass substrates [56,57] and the substrate was cooled using liquid nitrogen, reducing the temperature of the substrate to be around −140 °C. This kind of substrate has a high SERS performance, but compared with Nanorod substrate, there is still room for improvement. In contrast, the preparation of nanorods does not require more complicated parameter adjustment and equipment requirements. Therefore, it is well worth fabricating and verifying the SERS performance of AuNRs. At present, there are a few reports on the preparation of tilted AuNRs by the OAD process. Dikovska et al. have reported the Au nanorods substrate fabricated by pulsed laser deposition based on glancing angle deposition [58]. The Au nanorods with average lengths of 50~70 nm that have a diameter in the range of 10~20 nm exhibited a SERS enhancement factor of 10^5^, which is lower than the best SERS enhancements factor of nanorod, 10^8^, reported earlier using oblique angle deposition [9,59]. One of the possible reasons says there is no underlayer Au film deposited underneath the AuNRs. Zhao et al. found that underlayer film can probably generate additional hot spots between the film and nanorods, and substantially improve the SERS signal. They improved the enhancement factor of the AgNR substrate by a factor of around 10^3^ through depositing underlayer Ag film [60]. Suzuki et al. have reported AuNRs arrays aligned in line by a dynamic oblique deposition technique [54]. The enhancement factor of AuNR arrays is estimated to be 10^9^, which is as large as that of the Ag nanorod arrays. Before AuNRs were deposited, the SiO_2_ template layer should be prepared by the serial bideposition (SBD) technique in advance. Therefore, it is meaningful to study AuNRs that can be deposited in one-step and have a high SERS performance. 

To apply the SERS technology into a real-life area is a mission that lots of researchers have made great efforts to accomplish. Amphetamines, represented by methamphetamine, are the most widely used synthetic drugs. The yield and consumption increase year by year. This kind of drug has brought great harm to society, triggered various illegal and criminal activities, which have seriously disturbed public security. The rapid detection of drugs is of great significance for combating crime and suppressing the epidemic of drugs. At present, common methamphetamine detection methods include gas chromatography [61], high-performance liquid chromatography [62], gas chromatography-mass spectrometry [63], enzyme-linked immunosorbent assay [64], and the immunocolloid gold technique [65]. Among them, the first few methods have higher requirements for analysts and equipment, and they are not suitable for on-site testing. The latter two methods require expensive reagents. The LOD of the commercial colloidal gold test paper is 6.7 × 10^−6^ M. Sometimes false positive signals may be given. There is an urgent need for a reliable on-site rapid detection technology for amphetamines. SERS is a highly sensitive sensing technology with a single molecule “fingerprint” identification capability. With the development of portable Raman spectroscopy, SERS technology can play an important role in the field of drug detection.

In this work, aligned AuNRs arrays have been successfully fabricated for surface-enhanced Raman scattering based on OAD process. The EM filed distribution of AuNRs arrays was visualized via FDTD calculation. The length and tilting angle of AuNRs were optimized. On the basis of SEM images, the relationship between process parameters and structure parameters has been discussed for guiding the process. SERS performance was validated using BPE as the probe molecule. The LOD can reach 10^−11^ M. We tested methamphetamine with the AuNRs substrate. The LOD can reach 10^−7^ M (i.e., 14.92 pg/ml), which met the requirements for the on-site rapid detection of the methamphetamine in human urine (500 ng/ml). This is better than the LOD of the commercial colloidal gold test paper, i.e., 10^−6^ M. The AuNRs substrate provides a good platform for trace biochemical detection based on SERS.

## 2. Materials and Methods

### 2.1. Materials

1,2-bis (4-pyridyl) ethylene (BPE) was purchased from Sigma-Aldrich., USA. Silicon wafer purchased from the 42nd Institute of the fourth Academy of CASC was used as the deposition substrate. Gold (Au) pellets with the purity of 99.99% and chromium (Cr) pellets with the purity of 99.999% were purchased from Jinyu Aochen (Beijing, China). Ethanol (C_2_H_5_OH), sulfuric acid (H_2_SO_4_) and hydrogen peroxide solution (H_2_O_2_) were purchased from Sinopharm (Shanghai, China). Twice distilled water (resistance rate ≥18.2 MΩ∙cm) was used as the water sample.

### 2.2. Apparatus

AuNRs array was deposited on the cleaned silicon substrate in a custom modified e-beam evaporator (ZZS500, Nanguang, Chengdu, China). The Raman spectra were collected with a portable Raman system equipped with a 785 nm excitation source and the laser spot diameter was 106 (BWS415-785S, from B&W, Tek, Newark, DE, USA). The field-emission SEM (S-4800, Hitachi, Tokyo, Japan) was used to analyze the morphology of the film.

### 2.3. FDTD Modeling

**Figure 1:**
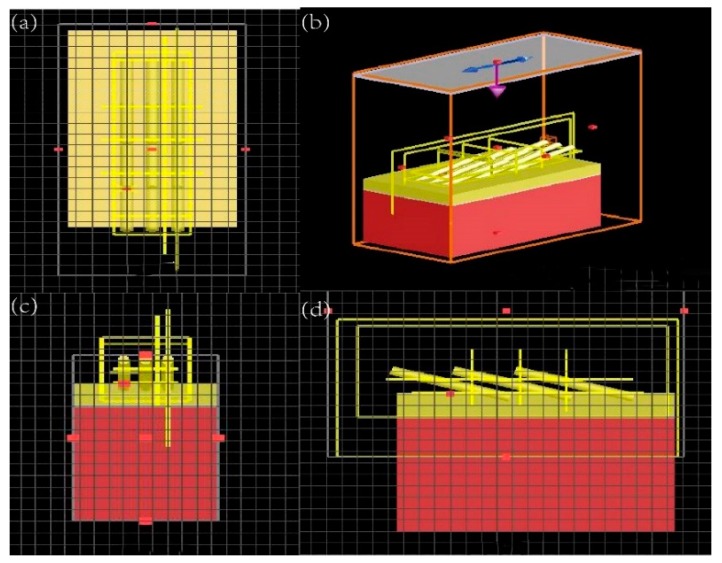
Models of Au nanorods (AuNRs) for finite-difference time-domain (FDTD) simulation: (**a**) XY plane, (**b**) oblique view, (**c**) XZ plane, (**d**) YZ plane.

FDTD (finite-difference time-domain) software was utilized to visualize the electromagnetic characteristic of the AuNRs. The simulation model was constructed using the 3D modeling function of the software, as shown in Figure 1. The Z-axis direction was defined as surface normal of the substrate. Y-axis direction was defined to be perpendicular to Z-axis direction and along the growing direction of AuNRs. X-axis direction was perpendicular to both Y-axis and Z-axis direction.

The size of the Si substrate model was 1000 nm × 1800 nm. The bottom layer of Cr on the Si substrate was 20 nm thick, and the layer of Au on the Cr was 100 nm thick. The Au nanorod distributed regularly on the Au layer. The length of AuNRs, L, was set to be 400, 600, 800, 1000, and 1200 nm and the tilting angle β was set to be 67°, 69°, 71°, 73°, 75°, and 77°, respectively. In order to simplify the model, only three rows and three columns of AuNRs were employed, the distance between two NRs along the X-axis was 550 nm and 150 nm along the Y-axis. The meshes were set with the cell size of 2 nm × 2 nm × 2 nm. The perfectly matched layer absorbing boundary conditions were adopted in the X, Y, Z-direction. The plane wave with a wavelength of 785 nm was utilized as the laser source which was normally incident to the substrate with the polarization parallel to the X-direction.

### 2.4. Preparation of AuNRs Substrate

**Figure 2:**
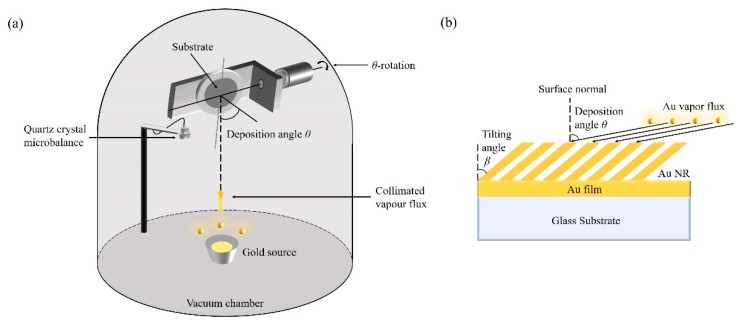
(**a**) The schematic of the Oblique Angle Deposition (OAD) process. (**b**) The schematic of the AuNRs structure. The definition of the deposition angle θ and the tilting angle β is shown.

The AuNRs substrate was fabricated by the OAD method. The schematic of the process is shown in Figure 2a. The deposition angle θ is defined as the angle between the substrate surface normal and vapor flux direction. Before deposition, all glass slides with a typical dimension of 10 mm × 10 mm and silicon (100) substrates with a typical dimension of 5 mm × 5 mm were cleaned in the Piranha solution, then rinsed with deionized water for three times and dried by nitrogen. A thin layer of 20 nm Cr was firstly deposited onto the cleaned substrate with deposition angle θ at 0°. The purpose of this step is to increase the adhesion of the gold film to substrate. Secondly, a plain layer of 100 nm Au was deposited onto the Cr thin film in order to enhance the adsorption of the columnar structure on the surface. This also can enhance the SERS performance of the AuNRs structure. Thirdly, AuNRs were prepared by the OAD technique with a big deposition angle (usually θ > 75°). During deposition, the thickness of the deposited film was monitored by a quartz crystal microbalance (QCM) positioned near the substrate. The pressure in the vacuum chamber was around 4 × 10^−4^ Pa. The deposition angle and deposition rate are the two key process parameters. By adjusting these two parameters, AuNRs with different structures can be obtained. Several deposition angles, θ = 80°, 82°, 84°, 86°, and 88°, and several deposition rates, v = 1, 2, 3 Å/s have been adopted during experiments in order to find the optimized process parameters.

Figure 2b shows the schematic of the fabricated AuNRs. The definition of the deposition angle θ and the tilting angle of AuNRs, β, are given. The principle of OAD is the shadowing effect during evaporation [66]. As shown in Figure 2b, β is always smaller than θ. The growth dynamics will determine the relationship between β and θ. This is also one of the important topics which this paper will talk about in detail.

### 2.5. Raman Measurements

Raman spectra were collected with the Raman microscope (innoRam, B&W Tek. Inc., Newark, DE, USA) equipped with a 785 nm laser source. Spectra were obtained for 10 s integration time, and the laser power was about 90 mW. The curves presented here were drawn according to the dark subtracted data and for the average from nine randomly chosen spots of a given substrate. BPE was chosen to be the probe molecule to characterize the SERS enhancement ability of the AuNRs.

## 3. Results and Discussion

### 3.1. FDTD Calculation

**Figure 3:**
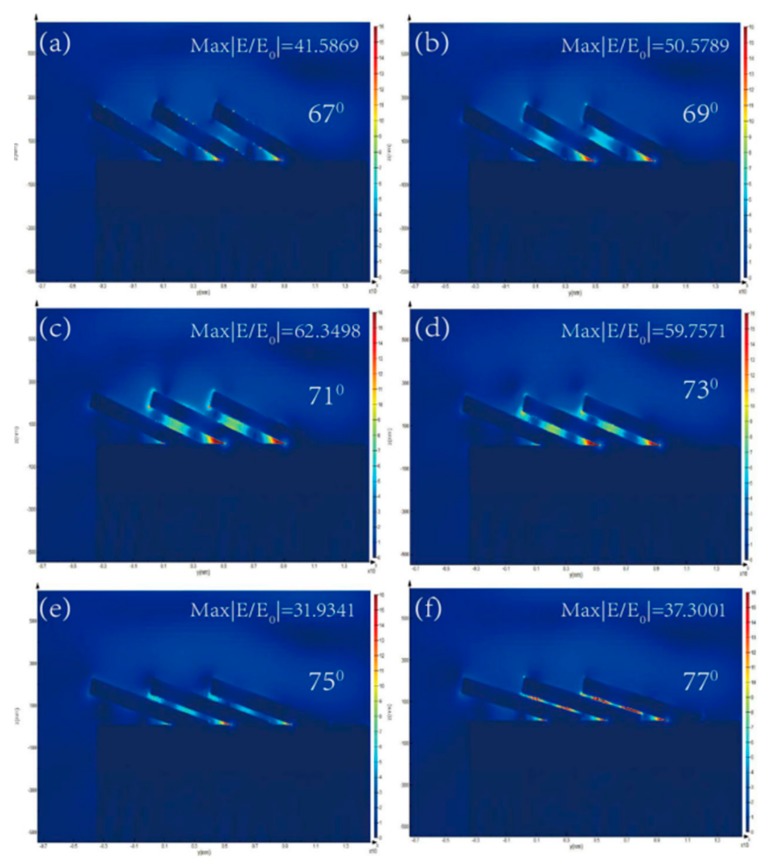
Calculation results of the AuNRs array with different tilting angles by the FDTD method: (**a**) β = 67°; (**b**) β = 69°; (**c**) β = 71°; (**d**) β = 73°; (**e**) β = 75°; (**f**) β = 77°.

**Figure 4:**
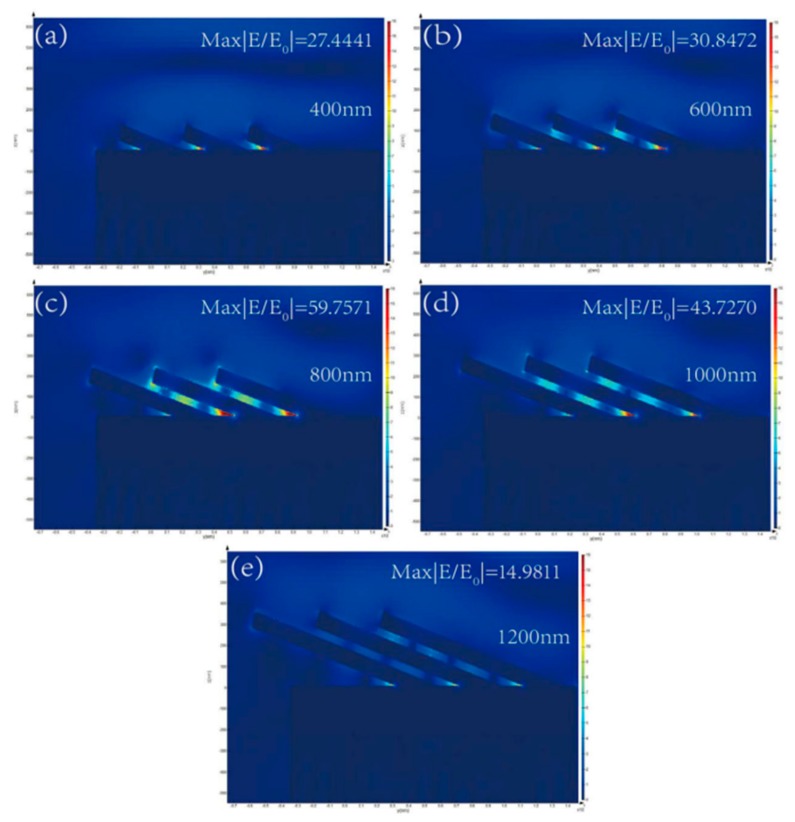
Calculation results of the AuNRs array with different lengths by the FDTD method: (**a**) L = 400 nm; (**b**) L = 600 nm; (**c**) L = 800 nm; (**d**) L = 1000 nm; (**e**) L = 1200 nm.

In order to demonstrate the effect of the tilting angle and length of the AuNRs, the distributions of the electromechanical field were visualized by the FDTD calculation. The model and simulation parameters are described in Section 2.3. The results of the calculation were shown in Figure 3 and Figure 4.

**Figure 5:**
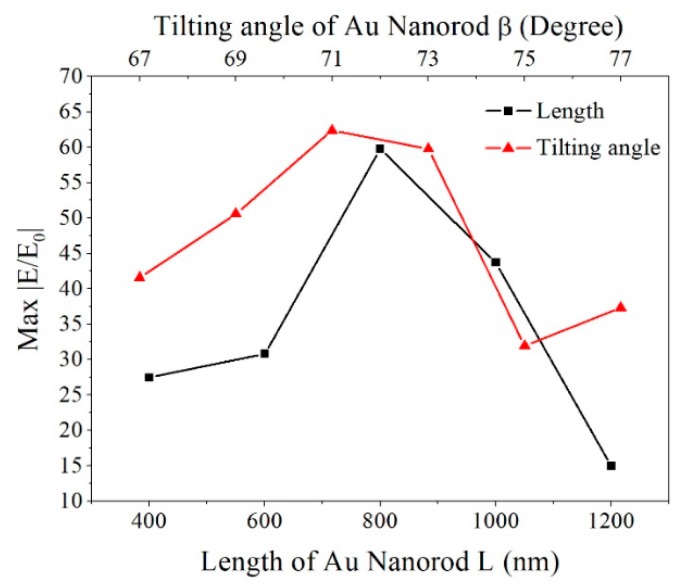
The electromagnetic field intensity as a function of AuNRs length and tilting angle.

Figure 3 shows the electromagnetic (EM) field distribution of the AuNRs array at different tilting angles. The EM field distributions are similar except for the magnitude of intensities. The “hotspots” are located at the connective position between AuNRs and the underlying Au film, the tip of nanorods and the gaps between the adjacent AuNRs. However, intensities of the “hotspots” vary with the different tilting angles. For tilting angle β = 67°, 69°, 71°, 73°, 75°, and 77°, the corresponding electromagnetic field intensity |E/E_0_| is 41.5869, 50.5789, 62.3498, 59.7571, 31.9341, and 37.3001. Figure 5 plots the electromagnetic field intensity of the Au nanorod array as the function of the tilting angle. With tilting angle of AuNRs increased from 67° to 71°, the electromagnetic field intensity gradually enhances. As the tilting angle increased from 71° to 77°, the electromagnetic field intensity gradually decreases. Considering the comprehensive factors, such as process and manufacturing cost, the tilting angle of the Au nanorod is optimized to be 71°.

Figure 4 shows the EM field distribution of the AuNRs array with different length. The “hotspots” distributions are also located at the same location as the simulation results shown in Figure 4. Intensities of the “hotspots” vary with different length of AuNRs. For length L = 400, 600, 800, 1000, and 1200 nm, the corresponding electromagnetic field intensity |E/E_0_| is 27.4441, 30.8472, 59.7571, 43.7270, and 14.9811, respectively. The electromagnetic field intensity of the Au nanorod array as the function of the length is also plotted in Figure 5. With the length of AuNRs increased from 400 nm to 800 nm, the electromagnetic field intensity gradually enhances. As length increased from 800 nm to 1200 nm, the electromagnetic field intensity gradually decreases. The electromagnetic field intensity reaches the maximum as the length of AuNRs is 800 nm. Also considering the comprehensive factors such as process and manufacturing cost, the length of the Au nanorod is optimized to be 800 nm.

### 3.2. Morphological Characterizations of Au Nanorod

**Figure 6:**
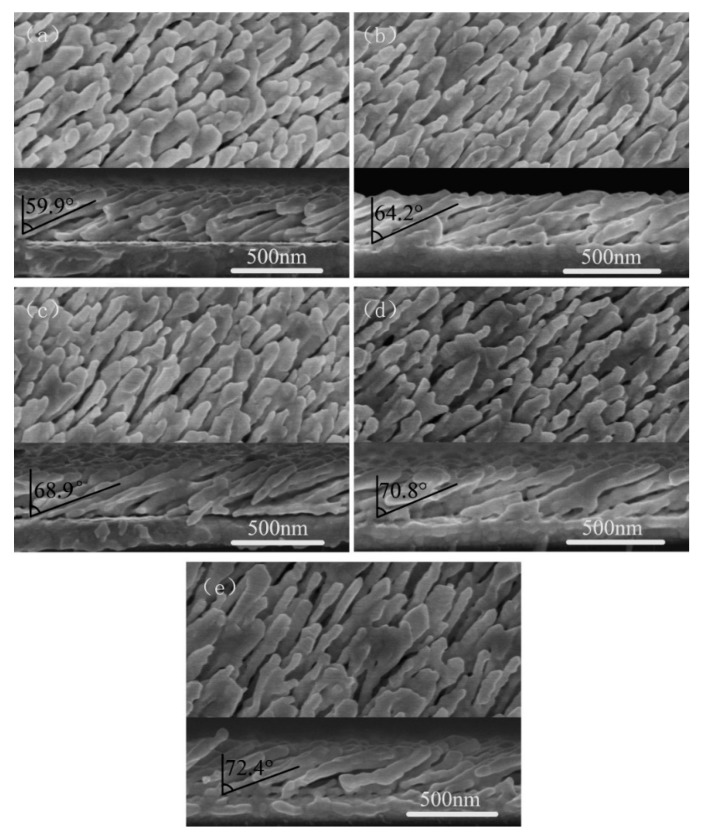
The SEM images of AuNRs deposited at different deposition angle θ: (**a**) θ = 80°, (**b**) θ = 82°, (**c**) θ = 84°, (**d**) θ = 86°, (**e**) θ = 88°. The upper part of each image is the top-view and the lower part is the cross-section view.

**Figure 7:**
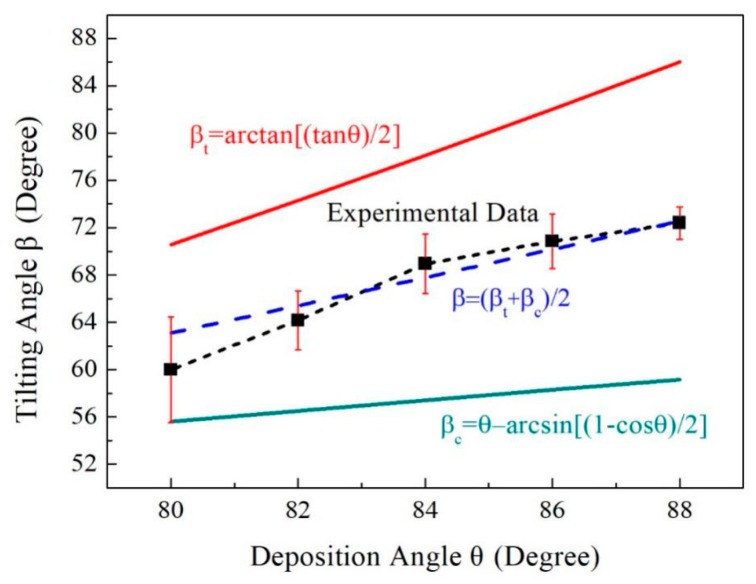
The Au nanorod tilting angle β as a function of deposition angle θ.

Top-view and cross-section SEM images of five representative Au nanorod arrays fabricated at deposition angle θ = 80°, 82°, 84°, 86°, and 88° are shown in Figure 6. In all experiments in this section, film thicknesses recorded by QCM were adjusted to make the length of AuNRs to be 800nm. The deposition rates were all set to be 1 Å/s. In Figure 6, we can see some nuances of morphologies of the five fabricated nanostructures. For small deposition angle, 80 and 82°, the Au nanorods tightly pack and fuse together. The diameter of the AuNRs is large and the distance between nanorods is small. With θ increased (θ=84, 86 and 88°), the gap between Au nanorods also increases. Hence the density decreases. The nanorod tilting angle β can be measured from the cross-section SEM images. Average tilting angles of five groups of nanorods with different deposition angles are shown in Figure 7. For θ = 80°, 82°, 84°, 86° and 88°, the corresponding tilting angle β are 59.9 ± 4.5, 64.2 ± 2.5, 68.9 ± 2.5, 70.8 ± 2.3, 72.4 ± 1.4°, respectively. The tilting angle β is always less than the deposition angle θ and increases with θ increased. The relationship between the tilting angle β and deposition angle θ is plotted in Figure 7. 

**Figure 8:**
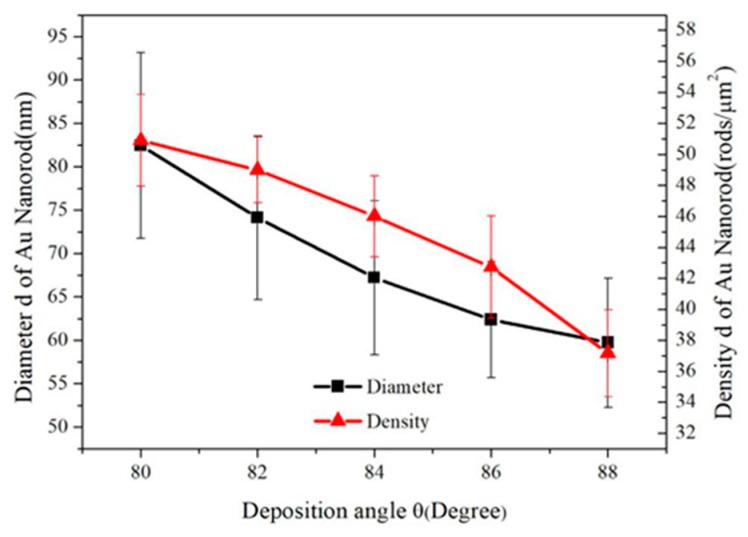
The plot of Au nanorod diameter and density vs. the deposition angle θ.

Different models describing the relationships between the tilting angle β and deposition angle θ have been proposed in the literature. The simplest and earliest relationship is the tangent rule, β_tangent_ = arctan(1/2tanθ), which is plotted using a red solid line in Figure 7. However, the tangent rule is not derived from a physical model and tends to overestimate β at a large deposition angle θ [67]. The relationship expressed by cosine rule, β_cosine_ = θ − arcsin[(1 − cosθ)/2], which is proposed based on geometric analysis [67], is plotted using the green solid line in Figure 7. The nanorod tilting angles predicted by cosine rule underestimate the true value. As shown in Figure 7, the true value is close to the average of angles calculated by tangent rule and cosine rule, i.e., β = (β_tangent_ + β_cosine_)/2, which is plotted in blue dashed line. This conclusion is consistent with the study of the previous literature [41]. The average diameters of nanorods are d = 82 ± 11, 74 ± 10, 67 ± 9, 62 ± 7, 60 ± 7 nm and the densities are 51 ± 3, 49 ± 2, 46 ± 3, 42 ± 3, 37± 3 rods/μm^2^, respectively. Figure 8 plots the density and the diameter of the Au nanorod as the function of the deposition angle θ. This trend is monotonic. The density and diameter will decrease as θ increased. Because of the increased deposition angle, the shadow effect will play a more important role during the evaporation. Hence the intercolumn void region expands, creating more room between columns and correspondingly reducing the density of the Au nanorods [66].

**Figure 9:**
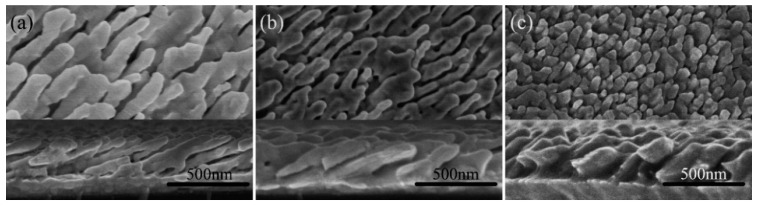
The SEM images of AuNRs deposited at different deposition rate R: (**a**) R = 1 Å/s, (**b**) R = 2 Å/s, (**c**) R = 3 Å/s.

SEM images of another three groups of Au nanorods are shown in Figure 9. The deposition rates are 1, 2, 3 Å/s, respectively. Film thicknesses recorded by QCM were adjusted to make the length of AuNRs to be 800 nm. The deposition angles θ are 86°. The density monotonically increases as the deposition rate increased. The average densities are 43 rods/μm^2^, 58 rods/μm^2^, 132 rods/μm^2^ for deposition rate R = 1, 2, 3 Å/s. For a small deposition rate (R = 1 Å/s, 2 Å/s), the gap distances between columns are 320 nm and 249 nm respectively. At these two deposition rates, rods are separated by void region due to the shadowing effect. For a fast deposition rate (R = 3 Å/s), the distance between columns is 105 nm, nanorods tightly pack with very small void regions. This result is also observed in ref. [68]. 

### 3.3. SERS Performance Characterization

To obtain the SERS enhancement ability of the AuNRs, the Raman spectra were acquired using BPE as the probe molecule. A series of concentrations of BPE ethanol solution ranging from to was prepared. BPE solution was dropped on the AuNRs substrates. Then the substrates were dried in atmospheric circumstance prior to measurement. 

**Figure 10:**
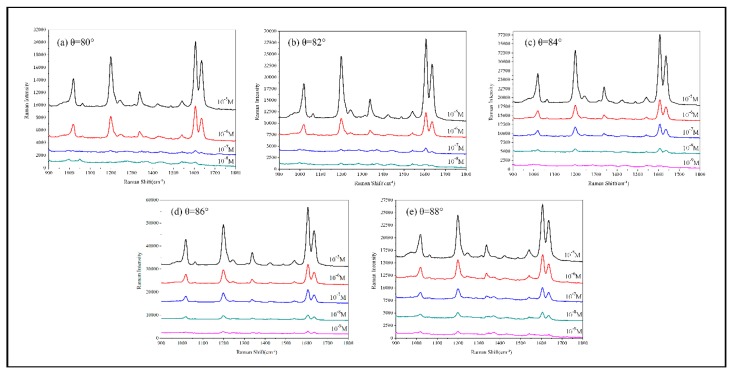
SERS spectra of BPE detected on the AuNRs substrates fabricated at different deposition angle: (**a**) θ = 80°; (**b**) θ = 82°; (**c**) θ = 84°; (**d**) θ = 86°; (**e**) θ = 88°.

Figure 10 shows the average SERS spectra of BPE detected on the AuNRs array substrates deposited at θ = 80°, 82°, 84°, 86° and 88°, respectively. All the SERS spectra show three characteristic peaks of BPE [69] listed in Table 2: (C=C stretching mode), (aromatic ring stretching mode) and (in-plane ring mode). Among the five substrates shown in Figure 6, the best LOD, 10^−9^ M, can be obtained for the nanostructures fabricated at deposition angle.

**Figure 11:**
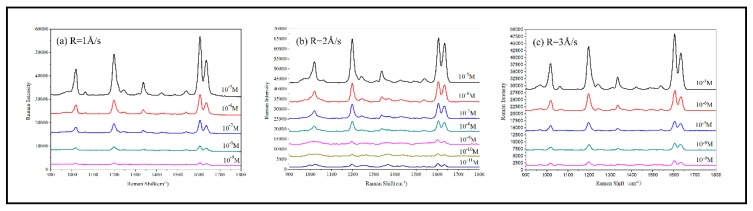
SERS spectra of BPE detected on the AuNRs substrates fabricated with different deposition rate: (**a**) R = 1 Å/s; (**b**) R = 2 Å/s; (**c**) R = 3 Å/s.

Figure 11 shows the SERS spectra of BPE detected on the AuNRs array substrates shown in Figure 9. Among these three substrates, the best LOD, 10^−11^ M, can be obtained with structures fabricated at deposition rate 2 Å/s.

In order to quantitatively compare the AuNRs substrates deposited at different deposition angles and deposition rates, the SERS peak at ∆ν = 1197.9 cm^−1^ and the equation EF = (I_SERS_/I_RS_) × (N_Vol_/N_Surf_) [70] were used to estimate the SERS enhancement factor (EF), where N_Surf_ = C_SERS_V_SERS_ × (S_laser_/S_SERS_) × N_A_ is the estimated number of absorbed molecules in the scattering volume for the SERS detection and N_Vol_ = C_RS_V_Laser_N_A_ is the estimated number of molecules in the scattering volume for the Raman (non-SERS) measurement. In our experiment, the normal Raman peak intensity of C_RS_ = 10^−2^ M BPE solution at ∆ν = 1197.9 cm^−1^ was I_RS_ = 32 counts. The volume of BPE solution producing Raman scattering was calculated to be V_Laser_ = 2.8 × 10^−12^ m^3^ [71] and thus, the amount of molecules excited in normal Raman measurement was estimated as N_Vol_ = 1.69 × 10^10^ (N_A_ = 6.02 × 10^23^ mol^−1^ is the Avogadro constant). In SERS measurement, V_SERS_ = 2 μl BPE solution which concentration is C_SERS_ = 10^−6^ M was dispersed onto the AuNRs substrate. In the actual experiment, the 2 μl BPE droplet is in fact spread over the entire 1 × 1 cm^2^ substrate, so S_SERS_ = 1 × 10^−4^ m^2^. The laser spot diameter is 106 μm and then the laser beam area S_laser_ = πr^2^ = 3.53 × 10 ^−12^ m^2^ on the substrate can be calculated. For different deposition angles θ = 80, 82, 84, 86 and 88°, the I_SERS_ = 3300, 3730, 3862, 6079 and 3953 counts, and EF = 4.10 × 10^7^, 4.62 × 10^7^, 4.79 × 10^7^, 7.53 × 10^7^ and 4.90 × 10^7^, respectively. The EF first increases with the deposition angle, reaches a maximum at the optimum deposition angle θ = 86°, and then decreases with further increase of deposition angle. For different deposition rates R = 1, 2, 3 Å/s, the I_SERS_ = 6079, 9239 and 5838 counts, and EF = 7.53 × 10^7^, 1.22 × 10^8^ and 7.24 × 10^7^. The computed results show that the EF reaches a maximum value when AuNRs substrate deposited at deposition angle θ = 86°, R = 2 Å/s.

Summarizing all the above-mentioned experiment data, we can get a conclusion that the best SERS enhancement performance can be obtained with the AuNRs deposited at angle 86° with rate 2 Å/s. The length of the AuNRs should be 800 nm.

### 3.4. SERS Detection of Methamphetamine

We chose the most sensitive AuNRs substrate with a length of 800 nm deposited at angle θ = 86° and rate R = 2 Å/s to detect methamphetamine. We first prepared a series of concentrations of methamphetamine solution ranging from 10^−3^ M to 10^−7^ M. 2 μl methamphetamine solution were dropped on the AuNRs substrates. Then the substrates were dried in atmospheric circumstance prior to measurement.

**Figure 12:**
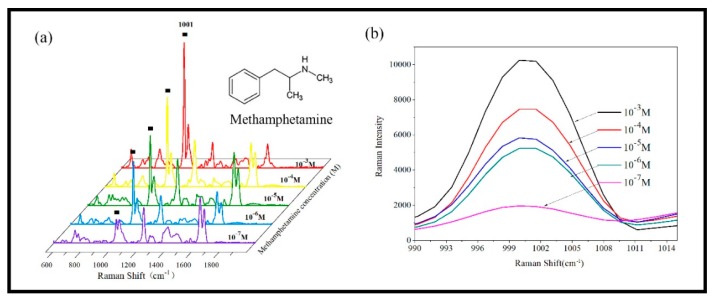
(**a**) The SERS spectra of AuNRs arrays substrate measured with various of methamphetamine; (**b**) the drawing of partial enlargement at the vibration bands of 1001 cm^−1^.

The characteristic peaks of methamphetamine are shown in Table 3. ∆ν = 620 cm^−1^, ∆ν = 1001 cm^−1^, ∆ν = 1021 cm^−1^ are attributed to the breathing vibration of the benzene ring. ∆ν = 1208 cm^−1^ is attributed to the phenyl-C stretching mode. ∆ν = 1582 cm^−1^, ∆ν = 1601 cm^−1^ are attributed to the stretching of the benzene ring. ∆ν = 835 cm^−1^ is attributed to the stretching vibration mode of isopropyl C-C. The spectra of different concentration detected are shown in Figure 12a. Among them, the characteristic peak of 1001 cm^−1^ has the highest intensity and is easier to be recognized. Therefore, the characteristic peak of 1001cm^−1^ is taken as a reference to study the variation of SERS signal intensity with the concentration of methamphetamine. Figure 12b shows the detailed changes in the SERS spectrum from 990 cm^−1^ to 1015 cm^−1^. As the concentration of methamphetamine decreased from 10^−1^ M to 10^−7^ M, the Raman intensity of the characteristic peak gradually decreases. The LOD is characterized to be 10^−7^ M (14.92 pg/ml), which meet the requirements of the on-site rapid detection of the methamphetamine in human urine (500 ng/ml). This is better than the LOD of the commercial colloidal gold test paper, i.e., 10^−6^ M. Moreover, the collection of SERS spectrum is seconds response. From sample preparation to test and then data analysis, the whole detection time was less than 5 min.

## 4. Conclusions

OAD is a very promising nanofabrication technique, which has the advantage of easy implementation, low cost, with no need for nanopattern, etc. To overcome the shortcomings of AgNRs as SERS substrate, such as easy to be oxidized and contaminated, we have proposed to fabricate AuNRs by the OAD process. FDTD simulation results show that, to obtain a maximum EM intensity, the optimized tilting angle of AuNRs should be 71° and the length should be 800 nm. SEM images show that tilting angle of AuNRs increased with the increase of the deposition angle from 80° to 88°, meanwhile, the diameter and density decrease. AuNRs with the tilting angle of 71° can be fabricated at deposition angle of 86°. When deposited at different deposition rates, the density of AuNRs increased with the increase of the deposition rate from 1 Å/s to 3 Å/s. Raman enhancement capability was validated by SERS measurement using BPE as the probe molecule. The best LOD was characterized to be 10^−11^ M using the substrate with length of 800 nm, which was fabricated at a deposition angle of 86° and deposition rate of 2 Å/s. This substrate was also used for the trace detection for methamphetamine. The LOD can reach 10^−7^ M (14.92 pg/ml), which meets the requirements of on-site rapid detection of methamphetamine in human urine (500 ng/ml). This result is better than the LOD of the commercial colloidal gold test paper, i.e., 10^−6^ M. Characterization results have shown that AuNRs fabricated by OAD is a very promising SERS substrate for trace analyte detection and bio-sensing.

## Figures and Tables

**Table 1:** Typical methods to fabricate solid-state surface-enhanced Raman scattering (SERS) substrates.

Method	SERS Performance	Repeatability	Uniformity	Substrate Area	Preparation Time	Preparation Cost
AAO [10,11,12]	high	good	good	wafer-level	short	lower
NSL [13,14,15,16,17,18,19,20,21,22,23,24,25,26]	higher	good	good	wafer-level	short	lower
EBL [27,28,29]	high	best	best	small	long	high
Self-Assembly [30,31,32,33,34,35,36,37]	high	best	best	wafer-level	short	low
OAD [38,39,40]	higher	best	best	wafer-level	shorter	lower

**Table 2:** Assignments of SERS bands of BPE.

Wavenumber (cm^−1^)	Assignments of SERS Bands
1198	C-C Stretch
1605	Aromatic ring stretching
1637	in-plane ring

**Table 3:** Assignments of SERS bands of methamphetamine.

Assignments of SERS Bands	Literature Report Wavenumber [72,73] (cm^−1^)	Experimental Data (cm^−1^)
the breathing vibration of the benzene ring	620	618
the stretching vibration mode of isopropyl C-C	834	835
the breathing vibration of the benzene ring	1001	1001
the breathing vibration of the benzene ring	1023	1027
the phenyl-C stretching mode	1207	1204
the phenyl-C stretching mode		1212
the stretching of benzene ring	1583	1580
the stretching of benzene ring	1601	1605
